# Interval Analysis-Based Optimization: A Robust Model for Intensity-Modulated Radiotherapy (IMRT)

**DOI:** 10.3390/cancers17030504

**Published:** 2025-02-03

**Authors:** Andrés Camilo Sevilla-Moreno, María Eugenia Puerta-Yepes, Niklas Wahl, Rafael Benito-Herce, Gonzalo Cabal-Arango

**Affiliations:** 1 School of Applied Sciences and Engineering, Universidad EAFIT, Medellín 050022, Colombia; mpuerta@eafit.edu.co; 2 Division of Medical Physics in Radiation Oncology, German Cancer Research Center, 69120 Heidelberg, Germany; n.wahl@dkfz-heidelberg.de; 3 Digital Health and Biomedical Technologies, Vicomtech Foundation, 20009 San Sebastian, Spain; rbenito@vicomtech.org; 4 Department of Radiation Therapy, Clínica El Rosario, Medellín 050012, Colombia; gacabal@clinicaelrosario.com

**Keywords:** radiotherapy, IMRT, uncertainty, robust optimization, interval analysis

## Abstract

**Background**: Cancer remains one of the leading causes of mortality worldwide, with radiotherapy playing a crucial role in its treatment. Intensity-modulated radiotherapy (IMRT) enables precise dose delivery to tumors while sparing healthy tissues. However, geometric uncertainties such as patient positioning errors and anatomical deformations can compromise treatment accuracy. Traditional methods use safety margins, which may lead to excessive irradiation of healthy organs or insufficient tumor coverage. Robust optimization techniques, such as minimax approaches, attempt to address these uncertainties but can result in overly conservative treatment plans. This study introduces an interval analysis-based optimization model for IMRT, offering a more flexible approach to uncertainty management. **Methods**: The proposed model represents geometric uncertainties using interval dose influence matrices and incorporates Bertoluzza’s metric to balance tumor coverage and organ-at-risk (OAR) protection. The θ parameter allows controlled robustness modulation. The model was implemented in matRad, an open-source treatment planning system, and evaluated on five prostate cancer cases. Results were compared against traditional Planning Target Volume (PTV) and minimax robust optimization approaches. **Results**: The interval-based model improved tumor coverage by 5.8% while reducing bladder dose by 4.2% compared to PTV. In contrast, minimax robust optimization improved tumor coverage by 25.8% but increased bladder dose by 23.2%. The interval-based approach provided a better balance between tumor coverage and OAR protection, demonstrating its potential to enhance treatment effectiveness without excessive conservatism. **Conclusions**: This study presents a novel framework for IMRT planning that improves uncertainty management through interval analysis. By allowing adjustable robustness modulation, the proposed model enables more personalized and clinically adaptable treatment plans. These findings highlight the potential of interval analysis as a powerful tool for optimizing radiotherapy outcomes, balancing treatment efficacy and patient safety.

## Simple Summary

Cancer treatment using radiotherapy aims to destroy tumors with high-energy radiation while minimizing damage to healthy tissues. However, small variations in patient positioning or internal organ movement can make treatments less effective or increase side effects. To manage these uncertainties, traditional methods add safety margins around the tumor, but this can lead to unnecessary exposure of healthy organs. Some advanced techniques focus on minimizing worst-case scenarios, but they can make treatments too conservative. In this study, we propose a new approach that represents these uncertainties as variable ranges rather than fixed values. This method, called interval analysis-based optimization, provides a more flexible way to balance treatment accuracy and safety. By testing this model on prostate cancer cases, we found that it improves tumor coverage while reducing radiation exposure to healthy tissues. This research contributes to more precise and personalized radiotherapy treatments, ultimately improving patient outcomes.

## 1. Introduction

Radiotherapy plays a fundamental role in the treatment of cancer, aiming to deliver high doses of ionizing radiation to tumors while minimizing exposure to surrounding healthy tissues. Advances in techniques such as intensity-modulated radiotherapy (IMRT) have allowed for more precise dose delivery, improving tumor control and reducing side effects. However, the effectiveness of IMRT is highly sensitive to geometric uncertainties, such as patient positioning errors and anatomical deformations, which can compromise the intended dose distribution.

Managing these uncertainties is essential to ensure that the prescribed radiation dose is accurately delivered to the tumor while minimizing exposure to nearby healthy tissue and organs at risk (OAR). The traditional approach to guaranteeing adequate dose coverage in photon radiotherapy, despite uncertainties, involves adding safety margins around the clinical target volume (CTV). These margins account for both systematic and random errors, as well as the internal motion of the target, to ensure that the CTV remains within the planning target volume (PTV) with high probability [1]. However, several studies have highlighted the limitations of this method, particularly in the context of IMRT.

A key issue is that the PTV concept, while simplifying treatment planning, does not sufficiently account for the complex, non-uniform nature of uncertainties, such as setup errors and organ motion. According to Unkelbach et al. [2], the use of rigid and uniform margins can lead to either over-coverage or under-coverage, compromising both treatment effectiveness and safety. When the CTV is near critical structures, overlap with the PTV may result in higher doses to healthy tissues, increasing the risk of side effects. Another significant limitation arises when the CTV is close to the skin, where there is little or no room to expand margins without risking excessive radiation dose to the skin or compromising target coverage [3]. This issue is particularly problematic in treatments involving superficial tumors, where precise dosing is critical to avoid complications.

On the other hand, during the early 21st century, several studies identified conceptual limitations of safety margins and the PTV approach in proton and ion beam therapy [4,5,6,7]. In response, alternative methods were developed. Cabal et al. [7] proposed a Monte Carlo-based iterative algorithm for non-isotropic volume expansion tailored to particle therapy. Robust optimization strategies were also introduced, integrating uncertainties directly into treatment planning by evaluating multiple error scenarios simultaneously, eliminating the need for PTV [2,8,9,10].

Among these methods, worst-case scenario or minimax approaches have gained the most adoption [8,11,12,13,14]. These strategies have shown success in proton therapy for clinical cases such as head and neck [11], lung [15], and oropharyngeal cancers [16], achieving excellent dose coverage and effective OAR sparing.

For photon beam therapy, however, the minimax approach, available in RayStation [17], has shown mixed results. Byrne et al. [18] found no notable benefits for breast cancer, while Archibald et al. [19] reported improved target coverage and fewer hot and cold spots. Exeli et al. [20] demonstrated better cerebral cortex sparing, and Zhang et al. [21] highlighted superior CTV coverage and reduced OAR doses in lung cancer compared to PTV-based methods.

In this context, interval analysis [22,23] provides a promising alternative to model and manage the possible geometric errors involved in the IMRT preparation and execution processes. Interval analysis has been widely applied in various fields of research, including engineering [24,25], portfolio [26,27,28], and control systems [29,30], where managing uncertainties and ensuring reliable solutions are critical. Its ability to represent uncertainties as bounded intervals, rather than relying solely on probabilistic assumptions, makes it a versatile tool for addressing complex optimization problems [31,32]. Despite its broad applications, its implementation in radiotherapy, particularly for managing uncertainties in dose optimization, remains unexplored. The novel contribution of this work lies in employing interval analysis to provide a continuous representation of uncertainties and using Bertoluzza’s metric to describe CTV objectives, offering a systematic and adaptable approach to improve tumor coverage and organ-at-risk sparing.

In this work, uncertain geometric parameters are represented as intervals, where each parameter is bounded by a lower and upper limit. For example, an interval [a,b] can describe the possible range of a patient’s positioning error during treatment delivery, with *a* representing the minimum displacement and *b* the maximum. This interval can also be expressed in terms of its center (nominal value) and radius (uncertainty or deviation from the nominal value), which is particularly useful in IMRT optimization as it separates the ideal, planned geometry from potential deviations caused by uncertainties.

One of the notable advantages of interval analysis in IMRT is its ability to ensure that treatment plans remain effective across the entire range of possible geometric uncertainties. Unlike minimax approaches, interval-based methods guarantee that solutions remain valid over a continuous variation of errors, not just in discrete worst-case scenarios [33]. This is crucial in IMRT, where even minor geometric deviations can lead to significant underdosing of the tumor or overdosing of healthy tissues.

Furthermore, interval analysis offers a systematic approach to error propagation [34,35], which can be effectively implemented in IMRT optimization. When multiple uncertain geometric parameters interact, such as patient shifts in different directions or the combined influence of various error sources, the resulting accumulated uncertainties can significantly impact the overall dose distribution. Interval analysis enables the computation of bounds on these interactions, ensuring that treatment plans comprehensively account for all potential variations in patient anatomy and positioning. By incorporating interval analysis, IMRT optimization can generate treatment plans that are not only robust against uncertainties but also reliable in delivering the prescribed dose to the target while sparing healthy tissues.

The proposed model was implemented in the open-source platform matRad [36] and evaluated using five clinical cases of prostate cancer. Results demonstrate that the interval-based approach achieves a superior balance between tumor coverage and OAR protection compared to traditional PTV and minimax models. These findings highlight the potential of interval analysis as a powerful tool for improving uncertainty management in radiotherapy, with implications for both clinical outcomes and personalized treatment planning.

## 2. Materials and Methods

### 2.1. The matRad Treatment Planning System

To implement the model and evaluate its results, we used matRad v3.1.0 [36], a treatment planning system created at the German Cancer Research Center (DKFZ). MatRad is an open-source project designed in MATLAB Version R2024b for research and educational purposes, featuring functions and classes that simulate the complete treatment planning workflow.

The routine used to minimize the objective function was Ipopt version 3.11.8, which is based on a primal-dual interior point method. The adaptive numerical differentiation tool DERIVESTsuite version 1.6 was used for gradient estimation in the minimax model. The calculations were executed on three identical nodes at the Apolo Scientific Computing Center of EAFIT University in Medellín, Colombia. Each machine has the following specifications: DELL PowerEdge M630 Server with an Intel Xeon E5-2683v4@2.10 GHz processor (32 cores), 64 GB DDR4 RAM @2400 MHz, 1 TB SATA III HDD @12 Gbps, and a high-speed network adapter: ConnectX-3 dual port.

### 2.2. Treatment Plan Evaluation Under Uncertainty (Robustness Perspective)

Evaluating the impact of uncertainties on treatment quality is not often part of treatment planning. Typically, errors are characterized before planning, and a margin recipe is applied based on clinical goals. Robust optimization requires evaluating plan robustness both during planning and for the final plan. We use three key concepts:

#### 2.2.1. Confidence Band Dose–Volume Histogram (cDVH)

A cDVH demonstrates the effect of uncertainties. The expected value and standard deviation of the volume points distribution for a given dose are computed based on scenario probabilities [37,38]. A dotted line shows the expected value, while a band around it, defined by one standard deviation, represents uncertainty. The nominal DVH is displayed as a solid line.

#### 2.2.2. Robustness Index

The robustness index (RI) quantifies CTV dose coverage under uncertain conditions. It is a novel methodology proposed in this work to evaluate the robustness of the treatments under geometric uncertainties. For each voxel in the CTV, we calculate:

(1) $$\Delta_{i}=\sqrt{{\left[\frac{E\left(d_{i}\right)-p}{c_{1}p}\right]}^{2}+{\left[\frac{std\left(d_{i}\right)}{c_{2}p}\right]}^{2}},$$

where $E(d_{i})$ and $std(d_{i})$ are the expected value and standard deviation of dose for voxel *i*, and *p* is the prescribed dose. Parameters $c_{1}$ and $c_{2}$ weight each component.

RI is calculated as:

(2) $$RI=\frac{|R_{CTV}|}{|V_{CTV}|}$$

where $|R_{CTV}|$ is the number of voxels with $\Delta_{i}<1$ and $|V_{CTV}|$ is the total number of CTV voxels.

#### 2.2.3. Price of Robustness

We build on the concept of the price of robustness, as described by Bertsimas et al. [39], which provides a theoretical framework for balancing robustness and optimality in decision-making under uncertainty. This concept highlights that ensuring feasibility and maintaining performance across a range of uncertain conditions often requires accepting solutions that deviate slightly from optimality in nominal scenarios.

The price of robustness is defined as the extent to which a solution diverges from the ideal nominal plan to account for uncertainties. It quantifies this trade-off by evaluating how much optimality is sacrificed to enhance the reliability and adaptability of the solution.

In any robust optimization model, this trade-off is inherent, as solutions must balance competing objectives: achieving robustness while minimizing deviations from nominal performance. Among models that achieve similar levels of robustness, those with the lowest price—the least deviation from nominal behavior—are preferred. This ensures that solutions are both practical and effective without introducing unnecessary conservatism.

In this study, the price of robustness is quantified by analyzing its impact on doses delivered to organs at risk (OARs) using the values of specific dosimetric metrics under the perfect positioning or nominal scenario. For prostate cancer cases, the metrics considered are:

- $V_{60Gy}$ [%] for the bladder, representing the percentage of bladder volume receiving a dose of 60 Gy or higher.
- $V_{40Gy}$ [%] for the rectum, representing the percentage of rectum volume receiving a dose of 40 Gy or higher.

This approach guarantees that the price of robustness is assessed using clinically significant dosimetric metrics specifically adapted to prostate cancer cases, enabling the selection of treatment plans that effectively balance robustness, safety, and treatment efficacy.

### 2.3. Interval Analysis-Based Model

In our approach, we utilize scenarios to explicitly evaluate and define the bounds of dose ranges under uncertain conditions. After establishing these boundaries, the model transitions from a discrete, scenario-based representation to a continuous interval-based description for dose values at each voxel. This transition allows the model to account for the inherently continuous nature of physical dose distributions, providing a finer and more accurate representation of uncertainties. By avoiding the discretization of distributions, the approach mitigates challenges such as the inability to capture subtle dose variations and the exclusion of intermediate scenarios not predefined in the discrete set. Furthermore, conventional robust approaches often rely on discrete probability distributions to model uncertainties. However, these methods are inherently limited by their dependence on predefined scenarios, which may fail to capture the full range of potential deviations, including less probable or intermediate variations. Interval analysis, on the other hand, considers uncertainties as continuous ranges within defined bounds, avoiding the need for specific probability assignments [22,40].

The foundational principles of interval analysis, as outlined by Moore [22] and Kearfott [23], emphasize the importance of range inclusion in optimization. By explicitly incorporating these bounds, the optimization accounts for all deviations within the interval, leading to solutions that are both reliable and conservative. The use of Bertoluzza’s metric [41] in this work is also crucial, as it facilitates the evaluation of both the midpoint and variability of dose distributions, enabling robust modeling of treatment plans while minimizing the effects of worst-case scenarios.

This approach bridges the gap between discrete and continuous perspectives by integrating interval theory with probabilistic insights. Unlike conventional methods that focus solely on expected or worst-case scenarios, the interval-valued approach considers the entire range of potential dose values, offering a more thorough evaluation [33].

#### 2.3.1. The Objective Function

The objective function of the interval-based model, denoted as $F_{INT}:X\to R^{+}$, incorporates Bertoluzza’s metric to account for various uncertainty sources. It is expressed as:

(3) $$\begin{array}{cc} F_{\mathrm{INT}}\left(x;\underline{\bar{D}},V,\theta \right) & =\omega_{\mathrm{CTV}}\underset{M.\mathrm{Bertoluzza}}{\underset{︸}{F_{\mathrm{CTV}}\left(x;\underline{\bar{D}},V_{\mathrm{CTV}},\theta \right)}}+\sum_{s\in S,s\neq \mathrm{CTV}}\omega_{s}\underset{\bar{d}\mathrm{penalties}}{\underset{︸}{F_{s}\left(x;\underline{\bar{D}},V_{s}\right)}} \end{array}$$

Here, $\underline{\bar{D}}=\left\{{\left(D\right)}_{c},{\left(D\right)}_{r}\right\}$ represents the set of dose influence matrices, with ${\left(D\right)}_{c}$ being the center dose matrix (representing the expected dose) and ${\left(D\right)}_{r}$ the radius dose matrix (capturing the standard deviation or variability of the dose). The optimization considers a set of voxels V, which includes structures S such as the clinical target volume (CTV) and organs at risk (OARs). Each structure *s* is assigned a weight $\omega_{s}$, adjusting its relative importance in the optimization process.

The term $\bar{d}$ denotes the upper bound of the dose interval, defined as:

$$\bar{d}={\left(d\right)}_{c}\left(x;\underline{\bar{D}}\right)+{\left(d\right)}_{r}\left(x;\underline{\bar{D}}\right),$$

where ${\left(d\right)}_{c}$ represents the expected dose and ${\left(d\right)}_{r}$ the standard deviation of the dose distribution for each voxel. This upper bound is utilized in penalty functions to quantify the impact of dose on OARs, ensuring that the optimization process identifies robust and conservative solutions to minimize exposure to healthy tissues.

By incorporating these elements, the objective function balances the competing priorities of tumor coverage and organ-at-risk protection, providing a framework to handle uncertainties systematically while maintaining clinically acceptable dose distributions.

#### 2.3.2. Bertoluzza’s Metric

Bertoluzza’s metric [41] is a valuable tool in error theory, particularly for analyzing uncertainties in fuzzy and interval data. Unlike traditional metrics such as the Hausdorff [42] or $L_{p}$ [43] metrics, it considers both midpoint and spreads of data, offering a more comprehensive representation of uncertainties. This dual focus makes it especially valuable when both the location and shape of uncertainties need to be assessed. By weighting the influence of the spread through a parameter, Bertoluzza’s metric enables a more nuanced analysis compared to simpler metrics, which may neglect the variability inherent in interval data.

In the context of IMRT optimization, we propose using Bertoluzza’s metric to directly incorporate geometric uncertainties into the problem formulation. A key aspect of this integration is the hyperparameter θ, which allows for flexible control over the balance between nominal accuracy and robustness to uncertainty. This flexibility ensures that treatment planning can adapt to varying clinical requirements, prioritizing either precision or robustness as needed.

Let $\underline{\bar{X}}=\left[x_{c},x_{r}\right]$ and $\underline{\bar{Y}}=\left[y_{c},y_{r}\right]$ represent intervals, where $x_{c},y_{c}$ are the interval midpoints, and $x_{r},y_{r}$ are the corresponding radii (spreads). The distance between these intervals, as defined by Bertoluzza’s metric, is given by:

(4) $$\begin{array}{cc} \left|\right|\Delta_{\theta }\left(\underline{\bar{X}},\underline{\bar{Y}}\right)\left|\right| & =\sqrt{{\left(x_{c}-y_{c}\right)}^{2}+\theta {\left(x_{r}-y_{r}\right)}^{2}},\;\theta \geq 0. \end{array}$$

This metric quantifies the combined effect of the differences in midpoints and spreads, with θ controlling the relative importance of the spread in the optimization.

For the interval-based optimization model, the objective function related to the clinical target volume (CTV) is expressed as:

(5) $$\begin{array}{c} F_{\mathrm{CTV}}\left(x;\underline{\bar{D}},V_{\mathrm{CTV}},\theta \right)=\sum_{i\;\in \;V_{\mathrm{CTV}}}{∥\Delta_{\theta }\left({\left[\underline{\bar{d}}\left(x;\underline{\bar{D}}\right)\right]}_{i},\underline{\bar{p}}\right)∥}^{2}, \end{array}$$

where:

$${\left[\underline{\bar{d}}\left(x;\underline{\bar{D}}\right)\right]}_{i}=\left[{\left[{\left(d\right)}_{c}\left(x;\underline{\bar{D}}\right)\right]}_{i},{\left[{\left(d\right)}_{r}\left(x;\underline{\bar{D}}\right)\right]}_{i}\right],\;\underline{\bar{p}}=\left[p,0\right].$$

Substituting these definitions, the objective function becomes:

(6) $$F_{\mathrm{CTV}}\left(x;\underline{\bar{D}},V_{\mathrm{CTV}},\theta \right)=\sum_{i\;\in \;V_{\mathrm{CTV}}}\left[{\left[{\left[{\left(d\right)}_{c}\left(x;\underline{\bar{D}}\right)\right]}_{i}-p\right]}^{2}+\theta {\left[{\left[{\left(d\right)}_{r}\left(x;\underline{\bar{D}}\right)\right]}_{i}\right]}^{2}\right],\;\theta \geq 0.$$

By adjusting θ, the model can prioritize either robustness to uncertainties or the nominal solution, offering a flexible and clinically relevant tool for radiotherapy treatment planning.

#### 2.3.3. Interval Dose Influence Matrices $\underline{\bar{D}}=\left\{{\left(D\right)}_{c},{\left(D\right)}_{r}\right)$}

In this framework, the dose interval $\underline{\bar{d}}\left(x\right)$ is defined as:

(7) $$\underline{\bar{d}}\left(x\right)=\left[{\left(d\right)}_{c}\left(x\right),{\left(d\right)}_{r}\left(x\right)\right],$$

where ${\left(d\right)}_{c}\left(x\right)$ represents the expected value of the dose distribution for each voxel across all scenarios, and ${\left(d\right)}_{r}\left(x\right)$ denotes the associated standard deviation, capturing the variability of the dose.

The center of the dose interval is calculated as:

(8) $${\left(d\right)}_{c}\left(x\right)=E\left[d(x;D_{k})\right]=\sum_{k\in K}w_{k}D_{k}x,$$

where the weights satisfy:

$$\sum_{k\in K}w_{k}=1.$$

Defining the center dose matrix as ${\left(D\right)}_{c}=\sum_{k\in K}w_{k}D_{k}$, Equation (8) simplifies to:

(9) $${\left(d\right)}_{c}\left(x;{\left(D\right)}_{c}\right)={\left(D\right)}_{c}x.$$

Similarly, the radius of the dose interval is computed as the standard deviation of the dose distribution:

(10) $$\begin{array}{c} {\left(d\right)}_{r}\left(x\right)=\mathrm{Std}\left[d(x;D_{k})\right]=\sqrt{\mathrm{Var}\left[d(x;D_{k})\right]}=\sqrt{E\left[d^{2}\left(x;D_{k}\right)\right]-{\left(E\left[d(x;D_{k})\right]\right)}^{2}}. \end{array}$$

Here, $E\left[d^{2}\left(x;D_{k}\right)\right]$ is calculated as:

(11) $$E\left[d^{2}\left(x;D_{k}\right)\right]=\sum_{k\in K}w_{k}d^{2}\left(x;D_{k}\right).$$

For each voxel *i*, the dose covariance matrix ${\left[{\left(D\right)}_{\mathrm{COV}}\right]}_{i}$ is expressed as:

(12) $${\left[{\left[{\left(D\right)}_{\mathrm{COV}}\right]}_{i}\right]}_{j_{1}j_{2}}=\sum_{k\in K}w_{k}D_{k,ij_{1}}D_{k,ij_{2}}.$$

The radius of the dose interval for voxel *i* can then be rewritten as:

(13) $${\left[{\left(d\right)}_{r}\right]}_{i}\left(x;{\left(D\right)}_{c},{\left(D\right)}_{r}\right)=\sqrt{x^{T}{\left[{\left(D\right)}_{r}\right]}_{i}x},$$

where:

$${\left[{\left(D\right)}_{r}\right]}_{i}={\left[{\left(D\right)}_{\mathrm{COV}}\right]}_{i}-{\left[{\left(D\right)}_{c}\right]}_{i}^{T}{\left[{\left(D\right)}_{c}\right]}_{i}.$$

The matrices ${\left(D\right)}_{c}$ and ${\left(D\right)}_{r}$ can be precomputed prior to optimization, simplifying computational demands during the iterative process.

The interval analysis-based optimization model offers a novel methodology in managing uncertainties and achieving a balance between tumor coverage and organ-at-risk protection. However, it requires greater computational resources compared to simpler models like PTV and minimax. The primary computational limitation arises from the memory requirements needed to store the covariance matrix for computing the dose radius matrix. Specifically, while the dose influence matrix for minimax models requires storing m×n×N elements (where *n* is the number of voxels in the geometry, *m* is the number of beam elements, and *N* is the number of stored scenarios), the interval-based model requires m×n elements for the center matrix and an additional $m^{2}\times n$ elements for the radius matrix. These increased memory demands can become significant for large-scale problems.

To address this issue, singular value decomposition (SVD) [44] is applied to reduce the size of the covariance matrix. By retaining only the *k* most significant singular values, the required memory is reduced to k×n+k×m+k, making the memory requirements manageable for computational resources commonly available in clinical radiotherapy settings.

#### 2.3.4. Singular Value Decomposition (SVD)

SVD is a mathematical technique that decomposes a matrix A of dimensions m×n into three matrices [44]:

$$A=U\Sigma V^{T},$$

where U is an m×m orthogonal matrix whose columns are the left singular vectors, Σ is an m×n diagonal matrix containing the singular values $\sigma_{i}$ in descending order, and V is an n×n orthogonal matrix whose columns are the right singular vectors. The singular values $\sigma_{i}$ represent the importance of the corresponding singular vectors in describing the variability or structure of A.

In this study, SVD is applied to the interval dose influence matrices $\underline{\bar{D}}$ to reduce the dimensionality of the problem. This reduction is critical for improving computational efficiency when handling large-scale matrices associated with dose distributions and uncertainties.

The decision to retain a subset of singular values is guided by the variance explained by the singular values. Mathematically, the proportion of variance explained by the first *k* singular values is given by:

$$\mathrm{Variance}\;\mathrm{Explained}=\frac{\sum_{i=1}^{k}\sigma_{i}^{2}}{\sum_{i=1}^{\mathrm{rank}(A)}\sigma_{i}^{2}}.$$

In this study, a threshold of 99% was applied to ensure that the retained singular values capture the majority of the matrix’s variability while effectively discarding noise and less significant components. For the evaluated prostate cases, retaining only the top *k* singular values, between 5 and 10, significantly reduced the storage and computational requirements for the matrices ${\left(D\right)}_{c}$ and ${\left(D\right)}_{r}$. This reduction enhances the scalability and efficiency of the interval-based model for large datasets without compromising its accuracy.

#### 2.3.5. The Hyperparameter θ

The parameter θ plays a critical role in the interval-based optimization model by modulating the balance between nominal dose accuracy and robustness against uncertainties. Introduced within Bertoluzza’s metric [41], θ weights the relative importance of deviations caused by uncertainties in dose distributions.

In the context of the optimization process, θ≥0 is a hyperparameter that adjusts the sensitivity of the model to dose variations. Mathematically, θ is integrated into the objective function as follows:

$$∥\Delta_{\theta }\left(X,Y\right)∥=\sqrt{{\left(x_{c}-y_{c}\right)}^{2}+\theta {\left(x_{r}-y_{r}\right)}^{2}},$$

where $x_{c}$ and $y_{c}$ are the nominal values (centers) of the intervals, and $x_{r}$ and $y_{r}$ represent their radii (uncertainties). A higher θ value increases the penalty for deviations due to uncertainties, emphasizing robustness, while a lower θ value prioritizes nominal accuracy.

The flexibility provided by θ is essential for tailoring treatment plans to individual patient needs. For example:

- When θ>1, the optimization focuses on robustness, making the solution less sensitive to significant deviations from the nominal scenario. This is particularly useful in cases where large uncertainties, such as patient motion, are expected.
- When 0≤θ<1, the optimization prioritizes accuracy in the expected (nominal) scenario, reducing the emphasis on extreme deviations. This is suitable when uncertainties are minimal or highly controlled.
- When θ=1, the model balances robustness and nominal accuracy equally, providing a middle ground between these two extremes.

The inclusion of θ in the optimization process provides a mechanism to systematically explore and adjust the trade-offs between treatment accuracy and safety. This adaptability makes the interval-based approach more versatile and applicable across a variety of clinical scenarios, enabling personalized treatment planning that accounts for both patient anatomy and sources of uncertainty.

#### 2.3.6. The Optimization Loop

**Figure 1:**
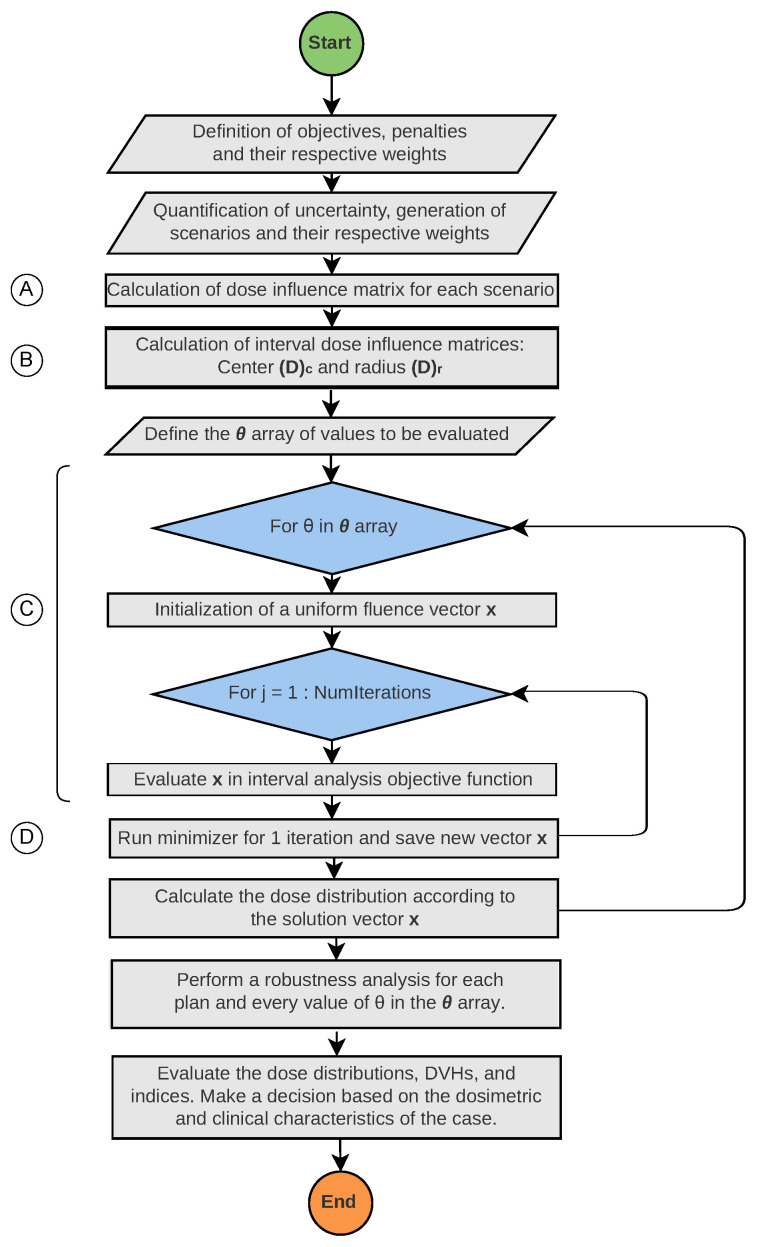
Interval-based optimization loop: The workflow highlights the most computationally demanding tasks in terms of resources and time: (A) calculation of dose influence matrices for each error scenario, (B) computation of dose interval matrices (center and radius), (C) fluence optimization, and (D) robustness evaluation.

Figure 1 presents a graphical sketch of the proposed methodology based on the proposed interval analysis-based optimization model.

### 2.4. Computational Study

We apply the interval analysis-based model to five prostate cases. For each of these cases, an IMRT plan is derived using a step and shoot technique. The beam angles used for the prostate cases were: 0°, 40°, 80°, 120°, 160°, 200°, 240°, 280°, and 320°.

The cases are identified with the prefix ‘P’ and the numbers ‘I’ to ‘V’ to differentiate each patient. Table 1 shows the IDs and, for preview purposes, the axial slices at the CTV geometric center:

Table 2 describes the complete set of objectives and penalties considered in the problem formulation. As a source of uncertainty, we chose set-up errors $\delta =\left(\delta_{x},\delta_{y},\delta_{z}\right)$ according to an uncorrelated 3D normal distribution function [45,46] $\mathrm{PDF}\left(\delta \right)=\mathrm{PDF}\left(\delta_{x},\delta_{y},\delta_{z}\right)$ with zero mean and standard deviation $\sigma =\left(\sigma_{x},\sigma_{y},\sigma_{z}\right)$, as follows:

(14) $$\mathrm{PDF}\left(\delta_{x},\delta_{y},\delta_{z}\right)=\frac{1}{{\left(2\pi \right)}^{3/2}\sigma_{x}\sigma_{y}\sigma_{z}}\exp\left(-\frac{1}{2}\left[\frac{\delta_{x}^{2}}{\sigma_{x}^{2}}+\frac{\delta_{y}^{2}}{\sigma_{y}^{2}}+\frac{\delta_{z}^{2}}{\sigma_{z}^{2}}\right]\right).$$

The standard deviations $\sigma_{x}$, $\sigma_{y}$, and $\sigma_{z}$ correspond to the directions LR, AP, and IS, respectively. The magnitude of the errors in patient positioning was set as σ=(5,10,5) [mm], based on a characterization of uncertainties conducted at Clinica El Rosario in Medellín, Colombia. This representation ensures a realistic approximation of geometric uncertainties observed in prostate cancer treatments.

Two sets of error scenarios were generated and used in this study, one for the optimization process and the second for treatment evaluation. For the optimization process, the set is composed of 13 scenarios: the nominal scenario and 12 shifting scenarios, 4 in each direction and a step size of 0.5σ. Using the dose influence matrix for all considered scenarios, the interval dose influence matrices (center and radius) were computed, describing the bounds of the contribution of each field element (bixel) to each geometry element (voxel).

For the evaluation, a grid of 5×5×5 scenarios centered on the perfect positioning scenario and a step size defined by 0.75σ was used. The vector *w* was calculated as described above.

For the treatment plans optimized under the planning target volume (PTV) approach, margins were applied in the left–right (LR), anterior–posterior (AP), and inferior–superior (IS) directions. The margins were (7, 7-4, 7) mm, where the margin was 7 mm in the anterior direction and 4 mm in the posterior direction.

## 3. Results

### Prostate Cases

**Figure 2:**
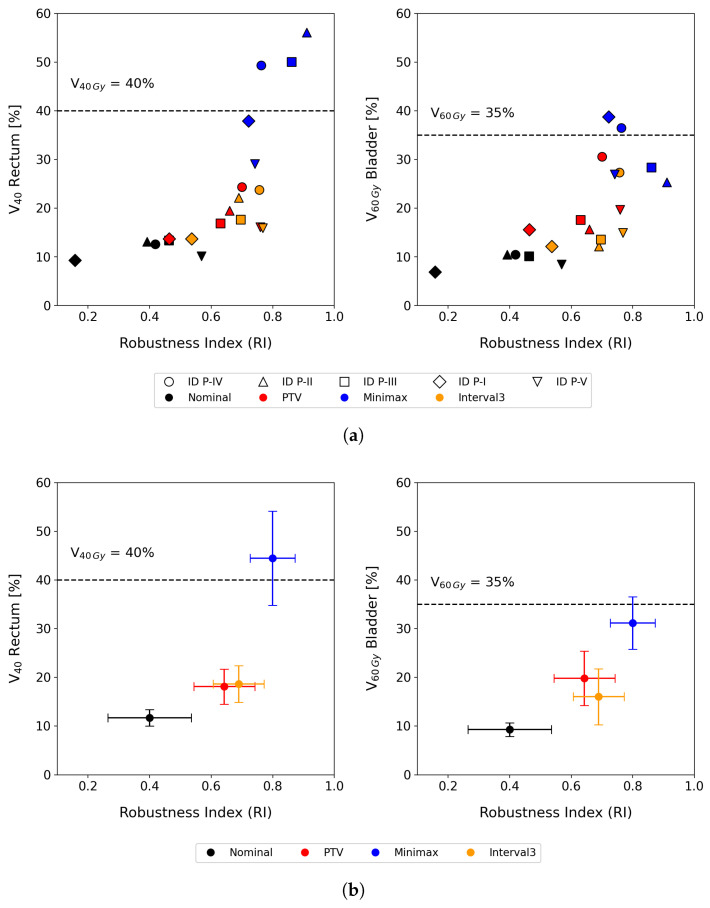
Price of robustness vs. robustness diagram corresponding to four treatment plans (nominal, PTV, minimax, and interval-based) for five different prostate cancer patients (ID P-I, P-II, P-III, P-IV, P-V). (**a**) Scatter plots representing the trade-off between robustness index (RI) and dose-volume metrics for the rectum ($V_{40\mathrm{Gy}}$) and bladder ($V_{60\mathrm{Gy}}$). Each marker corresponds to an individual patient, and different colors indicate different treatment plans. (**b**) Mean and standard deviation of the robustness index and dose-volume metrics across all patients for each optimization model. The dashed lines represent clinical threshold values for rectum and bladder dose constraints.

Figure 2 presents the results for five prostate cases, comparing the robustness indexes and the associated price in terms of dose to OARs for different optimization approaches: no uncertainty management (with the dose prescribed and optimized for the CTV only), safety margin (PTV), minimax model, and interval-based optimization. In the diagram, the horizontal axis indicates the robustness of the treatment plans, while the vertical axis represents the dose delivered to OARs. Ideally, a plan would have a robustness index of one and no dose contribution to healthy tissue, placing it in the lower right corner of the graph. Plans closer to this region are preferable as they strike a better balance, combining high robustness with minimal dose to OARs. In other words, these plans ensure patient safety while maintaining treatment efficacy.

**Figure 3:**
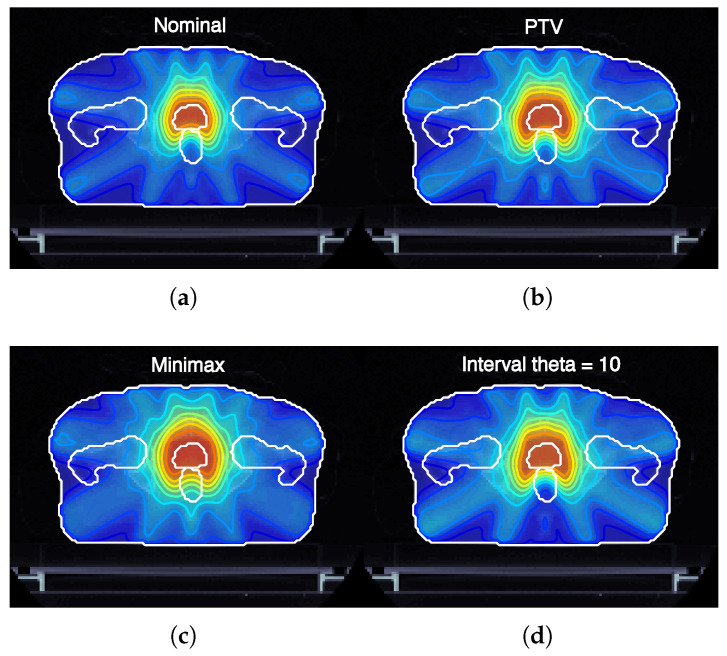
Expected dose maps (at isocenter) for prostate cancer plans, case ID P-I optimized using different treatment planning approaches. (**a**) Nominal plan without robustness considerations. (**b**) PTV-based plan incorporating margin expansion for geometric uncertainties. (**c**) Minimax robust optimization approach, aiming to minimize the worst-case scenario. (**d**) Interval-based robust optimization with θ=10, allowing for controlled robustness modulation.

In order to better understand the impact of the proposed model, we present below the detailed quantitative results for the patient with ID P-I. Figure 3 shows the expected dose map for the dose distribution resulting from each optimization strategy.

**Figure 4:**
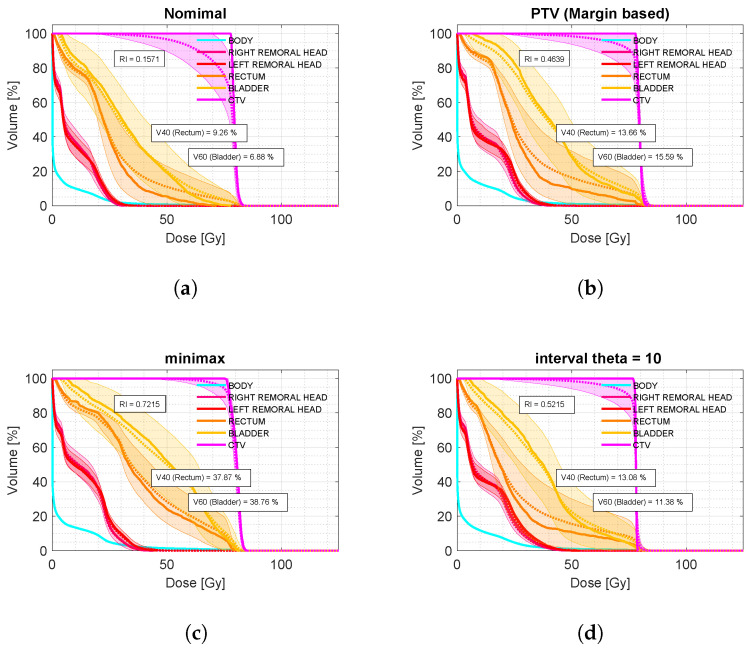
Dose volume histograms with confidence bands (cDVH) corresponding to four treatment plans for the prostate cancer patient ID P-I. (**a**) Nominal plan, optimized without robustness considerations, showing the best protection for organs at risk; however, the wider confidence band for the CTV indicates uncertainty in achieving the prescribed treatment dose within the tumor volume. (**b**) PTV-based plan, incorporating margin expansion to account for geometric uncertainties, leading to improved tumor volume coverage while slightly increasing the dose to organs at risk compared to the nominal plan. (**c**) Minimax robust optimization approach, which prioritizes worst-case scenario robustness, resulting in the best CTV coverage but over-irradiating healthy tissues, even exceeding the clinical constraint of V60 in the bladder. (**d**) Interval-based robust optimization with θ=10, providing the best balance between robustness and dose sparing, even surpassing the PTV model by improving coverage while reducing dose to organs at risk.

Figure 4 shows the confidence band DVH corresponding to results obtained with each model.

**Figure 5:**
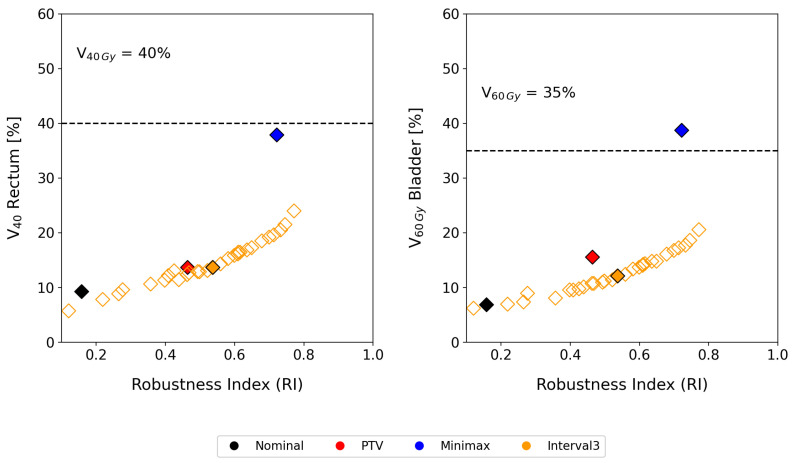
Price of robustness vs. robustness diagram corresponding to four treatment plans (nominal, PTV, minimax and interval) for the same prostate cancer patient ID P-I.

Figure 5 shows the correlation between robustness and the price of robustness, including, in the case of interval-based aproach, the results for every value of the parameter θ. The appropriate θ is based on evaluating the resulting dose distributions, similar to how different plans from various techniques are compared to choose the most suitable one for the patient. In this context, different levels of conservatism in incorporating uncertainties are compared. For this study, we selected the value of θ that produced plans with doses to OARs similar to those of the PTV approach, while maximizing robustness. However, this criterion should be established or modified by clinicians.

**Figure 6:**
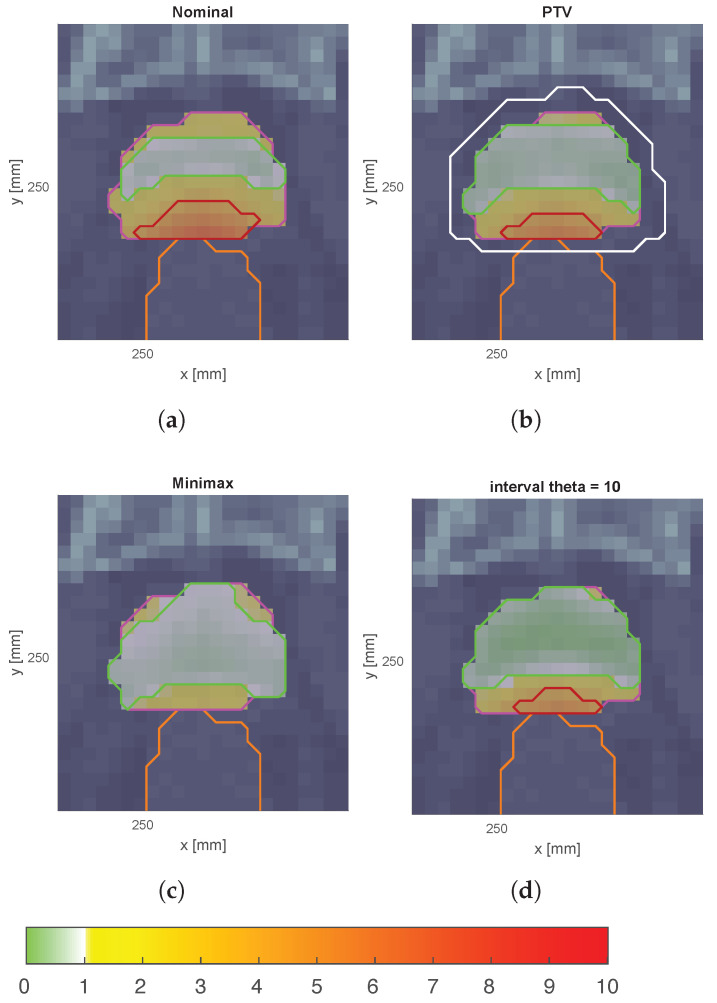
Δ-maps (at isocenter) for prostate cancer plans, case ID P-I optimized using different treatment planning approaches. The color scale represents the level of uncertainty in the prescribed dose deposition, where lower values (green) indicate minimal deviations and higher values (red) correspond to greater discrepancies. (**a**) Nominal plan, showing higher sensitivity to geometric uncertainties, particularly at the tumor edges. (**b**) PTV-based plan, improving robust dose coverage; however, maintaining a region of high uncertainty near the rectum. (**c**) Minimax robust optimization, showing the most extended dose coverage in the tumor volume. (**d**) Interval-based robust optimization, achieving a balance between uncertainty management and dose sparing, with improved tumor coverage compared to the PTV approach. (**d**) Interval-based robust optimization, achieving a more extended robust dose coverage than the PTV strategy, with the lowest voxel-wise uncertainty values among the four evaluated models.

Figure 6 shows the Δ-map for the dose distribution resulting from each optimization strategy. The voxels of CTV with a Δ-Value less than 1 (colored in green) are considered robustly treated according to the definition presented in Section 2.2.

Table 3 presents the values of robustness and the price of robustness for the P-I prostate treatment plans, as defined in Section 2. It is observed that tumor volume coverage, considering patient positioning errors, improves when robust approaches are used in treatment planning. The results obtained with the minimax and interval analysis models show better performance compared to the safety margin or PTV model, with increases of 25.8% and 5.8%, respectively, in the tumor volume receiving a robust dose.

In terms of the deposited dose in organs at risk, or considerations related to the price of robustness, the results were also better for plans optimized under interval analysis models. These plans showed a 4.2% reduction in the bladder volume covered by 60 Gy and a reduction in the rectum volume covered by 40 Gy. In contrast, the results for the minimax model were the least favorable, with increases of 23.3% for 60 Gy in the bladder and 24.2% for 40 Gy in the rectum compared to the PTV model.

## 4. Discussion

The results of this study demonstrate the effectiveness of incorporating interval analysis into the optimization of intensity-modulated radiotherapy (IMRT) treatment plans. By using an interval analysis-based model, we were able to achieve a more flexible and robust representation of uncertainties in patient positioning and the corresponding dose distribution. This method contrasts with traditional models, such as the planning target volume (PTV) approach and the minimax model, which either oversimplify the problem or overemphasize the impact of extreme deviations. Figure 3 illustrates the expected dose maps at the isocenter for prostate cancer plans (case ID P-I) optimized using nominal, margin-based, minimax, and interval-based models. Notably, the interval-based model achieves dose distributions adapted to the specific shape and magnitude of each type of uncertainty considered. This adaptability is achieved by optimizing the dose regionally, expanding it in areas where uncertainties are likely to compromise target coverage and reducing it in regions where healthy tissue sparing is critical. Such a strategy ensures that treatment objectives, including robust tumor coverage and organ-at-risk protection, are met more effectively than with conventional models.

Compared to the conventional PTV model, our interval analysis approach consistently provided better tumor coverage while minimizing the dose to organs at risk (OARs). As shown in Figure 2 (bottom), for the analyzed sample of prostate cases, the interval analysis and minimax models led to improvements of 5.8% and 25.8% in robust tumor coverage compared to the margin-based model. In terms of OAR dose, the interval-based model achieved a 4.2% reduction in $V_{60}$ for the bladder while maintaining the same $V_{40}$ value for the rectum. In contrast, the minimax model resulted in a 23.2% increase in $V_{60}$ for the bladder and a 24.2% increase in $V_{40}$ for the rectum compared to the margin-based strategy.

Despite these promising results, what distinguishes our method is its ability to properly modulate both robustness and the price of robustness. As shown by the results for patient P-1 in Figure 5, tumor coverage improved by 5.8% using interval analysis and by 25.8% with the minimax model compared to the margin-based approach. Nevertheless, the minimax approach resulted in an overly conservative treatment plan. In contrast, the interval method offered a more balanced compromise between robustness and OAR dosage. Even if the clinical decision for treatment selection prioritizes tumor coverage, by selecting an appropriate θ value, it is possible to achieve dose coverage values as high as 0.79, surpassing even the minimax model, while maintaining $V_{60\mathrm{Gy}}$ in the bladder and $V_{40\mathrm{Gy}}$ in the rectum below 20%.

### 4.1. Clinical Impact of Robustness Modulation Through the θ Parameter

One of the key strengths of the interval-based approach is its flexibility in managing uncertainties. By introducing the parameter, the model allows clinicians to modulate sensitivity to geometric uncertainties and tailor treatment plans to individual patients. This adaptability is particularly critical in cases involving significant anatomical variability or when tumors are located near critical structures, requiring a careful balance between robustness and accuracy.

Figure 5 illustrates how varying the θ parameter impacts the balance between robustness and organ-at-risk (OAR) protection. Increasing θ enhances the robustness of the treatment plan, ensuring that tumor coverage remains consistent even under significant uncertainties. Conversely, lowering θ prioritizes OAR sparing, reducing the dose delivered to healthy tissues. This dynamic modulation provides clinicians with a powerful tool to fine-tune treatment plans based on the specific clinical priorities of each patient, whether it is minimizing toxicity or ensuring robust tumor control.

Compared to conventional models, such as the planning target volume (PTV) approach or minimax optimization, the interval-based model demonstrates clear advantages. It achieves superior tumor coverage under uncertain conditions, leading to improved disease control. Simultaneously, it reduces doses to organs at risk (OARs), resulting in less toxic treatments with fewer complications and side effects. This dual benefit enhances the efficacy of radiotherapy while improving patient quality of life by minimizing treatment-related toxicity.

While this study primarily addressed uncertainties in patient positioning, the interval analysis-based optimization model is versatile and can be extended to other sources of uncertainty, such as:

- Inter-fractional variations: changes in organ position and shape between treatment sessions, which can accumulate over the course of radiotherapy.
- Intra-fractional motion: real-time organ motion during dose delivery, such as respiratory-induced movement in lung cancers or bladder filling.
- Tumor evolution: progressive changes in tumor size and shape during treatment, requiring adaptive re-planning.

The findings of this study highlight significant clinical implications. Integrating interval analysis into IMRT planning offers a comprehensive representation of dose distribution and associated uncertainties. This approach is particularly beneficial for tumors near sensitive structures, as it enables precise sparing of healthy tissue while maintaining effective tumor coverage.

### 4.2. Future Directions

The interval analysis-based optimization model presents a promising framework for addressing uncertainties in radiotherapy. While the findings of this study are encouraging, there are several opportunities to further develop and expand the applicability of the model. This section identifies key areas for future research:

#### 4.2.1. Expanding the Model to Other Cancer Types and Uncertainty Sources

The interval analysis-based optimization model, while evaluated in this study for prostate cancer cases, has the potential to be applied to other cancer sites where geometric and anatomical uncertainties significantly impact treatment planning. For example:

- Head and neck: The high variability in patient anatomy due to weight loss or tumor shrinkage makes head and neck cancers a suitable candidate for interval-based optimization. The model’s ability to account for continuous ranges of uncertainties could enhance dose delivery accuracy while sparing critical structures like the spinal cord or salivary glands.
- Lung: Respiratory motion presents a significant challenge in lung cancer treatments. By integrating four-dimensional computed tomography (4D-CT) imaging, the interval model can address uncertainties associated with breathing cycles, ensuring robust tumor coverage and minimizing the dose to healthy lung tissue.
- Pediatric: Pediatric patients often experience rapid anatomical changes during treatment. The interval-based approach can be extended to incorporate inter-fractional uncertainties, such as growth and organ deformation, enabling adaptive planning that evolves with the patient’s anatomy.

#### 4.2.2. Computational Efficiency of the Interval-Based Model

While the interval analysis-based optimization model demonstrates significant advantages in managing uncertainties in radiotherapy, its computational demands remain a challenge, particularly regarding memory demands in large-scale clinical applications. To address these limitations, several strategies can be explored to enhance computational efficiency without compromising accuracy.

The use of graphics processing units (GPUs) can dramatically speed up computationally intensive tasks:

- Matrix operations: GPU-accelerated libraries, such as CUDA or OpenCL, can handle large matrix operations, including the computation of interval dose influence matrices and singular value decomposition (SVD), at much higher speeds compared to CPU-based implementations.
- Parallelized dose calculations: Many steps in the dose calculation process, such as voxel-level dose contributions from multiple beams, can be parallelized and executed simultaneously on GPUs, reducing execution time significantly.

Beyond SVD, additional dimensionality reduction methods, such as principal component analysis (PCA) or tensor decomposition, could further reduce the computational burden by identifying and retaining only the most critical components of the dose influence matrices.

Future research should also focus on incorporating artificial intelligence (AI)-driven models to predict anatomical changes and automatically update interval bounds, further enhancing the adaptability of the model. Comparative studies assessing these enhancements and their impact on clinical accuracy are essential to ensuring that these methods maintain robustness without adding unnecessary complexity to treatment planning workflows.

Collaborative efforts between computational scientists and clinicians will be key to tailoring these innovations for practical use in radiotherapy.

#### 4.2.3. Retrospective Analysis for Clinical Impact Assessment

To validate the clinical potential of the interval-based model, retrospective analyses of previously treated patients using conventional methods could provide valuable insights. Such studies would involve the following steps:

1. Recalculation of treatment plans: Archived patient data, including CT images and dose distributions, can be used to recalculate treatment plans under the interval-based model. This would allow a direct comparison of dose metrics, such as tumor coverage and dose to organs at risk (OARs), across different optimization strategies (e.g., PTV, minimax, and interval-based).
2. Evaluation of robustness and price of robustness: Robustness index and the price of robustness could be computed for each recalculated plan, highlighting the ability of the interval-based model to balance treatment efficacy and safety under uncertainty.
3. Clinical outcome correlation: Patient outcomes, such as tumor control probability (TCP) and normal tissue complication probability (NTCP), could be retrospectively correlated with robustness metrics. This analysis would help determine whether the interval-based model translates into measurable clinical benefits, such as reduced toxicity or improved tumor control.

Such retrospective studies would serve as a foundation for prospective clinical trials, further validating the interval-based model and supporting its integration into routine radiotherapy workflows.

## 5. Conclusions

This study demonstrates that the incorporation of interval analysis into the optimization of intensity-modulated radiotherapy (IMRT) treatment plans offers significant advantages in managing uncertainties related to patient positioning. By employing interval dose influence matrices, our approach not only improves tumor coverage but also reduces the dose to organs at risk (OARs), providing a more balanced and flexible solution compared to traditional models such as the planning target volume (PTV) and minimax approaches.

One of the key contributions of this work is the ability to adjust the balance between robustness and the price of robustness using the θ parameter. This flexibility allows clinicians to fine-tune treatment plans according to the specific needs of each patient, ensuring more personalized and effective radiotherapy. In particular, our results show that, for prostate cancer cases, the interval analysis model was able to improve tumor coverage while reducing unnecessary dose exposure to OARs, offering better outcomes than either the margin-based or minimax strategies.

The adaptability of the interval-based approach also makes it applicable to a wide range of uncertainties beyond patient positioning. The method can be extended to other sources of uncertainty such as inter-fraction errors or anatomical changes over time, making it a versatile tool in clinical radiotherapy settings. For example, intra-fraction organ motion could be effectively modeled using 4D tomographic imaging [47], while inter-fractional variations could be represented through principal component analysis of patient data [48,49]. Moreover, with ongoing advances in computational technology, the current limitations related to memory and processing requirements are likely to become less of an issue, paving the way for wider adoption of interval analysis in clinical practice.

In conclusion, the use of interval analysis in IMRT treatment planning represents a promising direction for improving both the precision and adaptability of radiotherapy. By providing a more robust approach to uncertainty management, this method has the potential to enhance patient outcomes, particularly in complex cases where tumor control and normal tissue sparing are both critical. Future research should focus on further optimizing the computational efficiency of the model and exploring its application to different types of cancer and uncertainty sources, with the goal of establishing interval analysis as a standard tool in personalized radiotherapy.

## Figures and Tables

**Table 1:** Identification and preview of the prostate cases used in this study.

P-I	P-II	P-III	P-IV	P-V
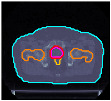	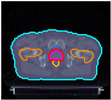	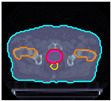	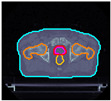	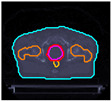

**Table 2:** Objectives and penalties used for treatment planning. PTV objectives marked with (*) were only used in the margin-based approach. For the nominal, minimax, and interval analysis models, these objectives were not taken into account.

Case	Structure	Type	Description	Weight
Prostate	CTV	Dose Min.	78 Gy	30
	CTV	SqDev	78 Gy	0.01
	CTV	MaxDVH	81 Gy<5%	10
	CTV	Dose Max.	83.5 Gy	100
	PTV *	Dose Min.	78 Gy	30
	PTV *	MaxDVH	81 Gy<5%	10
	PTV *	Dose Max.	83.5 Gy	100
	BODY	SqOver	39 Gy	10
	Ring 0–20 mm	MaxDVH	83.5 Gy	100
	Ring 20–50 mm	MaxDVH	78 Gy	100
	Bladder	MaxDVH	$V_{60Gy}<\mathrm{dose}\;\mathrm{puuling}\;\mathrm{result}$	2
	Rectum	MaxDVH	$V_{40Gy}<\mathrm{dose}\;\mathrm{pulling}\;\mathrm{result}$	2

**Table 3:** Robustness and price of robustness evaluation for prostate cancer clinical case ID P-I.

Model	RI	$V_{40\mathrm{Gy}}$ Rectum [%]	$V_{60\mathrm{Gy}}$ Bladder [%]
Nominal (CTV)	0.157	9.3	6.9
PTV	0.464	13.7	15.6
Minimax	0.722	37.9	38.8
interval-based	0.522	13.1	11.4

## Data Availability

All relevant data are contained within the article. The complete code used for the model implementation is available at https://github.com/acsevillam/matRad/tree/cminimax2 (accessed on 25 January 2025).
